# Point of care ultrasound - a way to reduce radiation exposure of patients and medical staff

**DOI:** 10.1186/2197-425X-3-S1-A273

**Published:** 2015-10-01

**Authors:** T Zawada, A Wieczorek, P Garba

**Affiliations:** 4 WSK z Poliklinika we Wroclawiu, Wrocław, Poland

## Introduction

Lungs ultrasonography and echocardiography is used by intensivist to provide assessments in patients with significant respiratory and cardiac diseases.

Transthoracic lung ultrasound allows to detect lung aerations and consolidations, to find pneumothorax or hydrothorax, localize alveolar-interstitial edema. It is also used as a monitoring tool to detect reaeration resulting from efficient treatment of pneumonia and lung recruitment resulting from PEEP. All the ultrasonographic measurements are made noninvasively and at the patient's bedside. Lung ultrasonography has been performed in our ICU for three years. Since then a tendency to reduce number of chest x-rays and chest CT has been observed.

## Objectives

The aim of the study was to analyze whether usage of the lung utlrasonography as a diagnostic and monitoring tool leads to minimize the number of chest x-rays and chest CT and radiation dose in the ICU.

## Methods

Data from 2 years before and 2 years after implementing a routine lung ultrasonography have been compared and statistical analysis has been made.

## Results

**Table 1:** Table 1

	**2011-2012**	**2013-2014**	**p**
Number of hospitalized patients	666	648	NS
SAPS2	52,6	53,0	NS
Mean time of ICU hospitalization (days)	7,1	8,0	NS
Number of chest x-rays	1744	1612	NS
NUMBER OF CHEST-CT	52	34	p < 0,05
SUMMARISE DOSE OF RADIATION/YEAR [mGY/1,70 m2 BSA]	1199,2	925,6	p < 0,05
Mean time of mechanical ventilation	6,2	8,3	NS
Mortality	36,5%	36,0%	NS

## Conclusions

The ability to perform transthoracic lung ultrasonography allowed us to reduce number of chest x-rays, chest-CT and overall radiation dose. The implementation of new diagnostic technique did not influence on length of hospital stay or mortality.

## Grant Acknowledgment

ICU Staff
